# The complete mitochondrial genome of *Apolemichthys trimaculatus* (Perciformes, Chaetodontidae)

**DOI:** 10.1080/23802359.2022.2054734

**Published:** 2022-07-25

**Authors:** Jin Gao, Hongji Ke, Wei Tan, Yongbo Wang, Chuan Lin

**Affiliations:** a Key Laboratory of Utilization and Conservation for Tropical Marine Bioresources (Hainan Tropical Ocean University), Ministry of Education, Sanya, China; b Hainan Provincial Key Laboratory of Tropical Maricultural Technologies, Hainan Academy of Ocean and Fisheries Sciences, Haikou, China; c Department of Animal Cultivation, Hainan Agriculture School, Haikou, China

**Keywords:** Mitogenome, *Apolemichthys trimaculatus*, phylogeny

## Abstract

Three-spot angelfish (*Apolemichthys trimaculatus*) is one of the most widespread angelfish that belongs to Pomacanthidae. However, there are few reports of the systemically classification and evolutionary analysis for *A. trimaculatus* so far. In this study, the complete mitochondrial genome of the *A. trimaculatus* is described. The full length of the mitogenome is 16,548 bp, consisting of 13 protein-coding genes, 22 transfer RNAs, two ribosomal RNAs genes, and a non-coding control region. The overall base composition is 28.4% for A, 25.6% for T, 29.5% for C, and 16.5% for G, with a slight AT bias (54.0%). The mitogenome of *A. trimaculatus* provided essential and valuable DNA molecular information for further phylogeny and management of angelfish species.

The three-spot angelfish *Apolemichthys trimaculatus* (Cuvier, 1831), belonging to the family of Pomacanthidae, was also known as Flagfin angelfish. The *A. trimaculatus* have a very distinctive bright coloration and blue lips, found in Indian Ocean and tropical western Pacific Ocean (Kuiter 1992). The species inhabits tropical regions and in rocky caves. In this study, the complete mitogenome of *A. trimaculatus* was determined with next-generation sequencing method. Although the phylogenetic relationships of subfamily Pomacanthinae had long been recognized and extensively studied (Shen, Chang, et al. 2015; Shen, Chen, et al. 2015; Yang et al. 2020), the molecular information was also expected to contribute identification and classification to the species more correctly. Elucidating the sequence and structure of *A. trimaculatus* mitogenome is useful for understanding the population genetics and molecular phylogeny of angelfish species.

An individual specimen of *A. trimaculatus* was collected from the sea area of Sansha (geographic location: 16°48′45″N, 111°83′82″E) of Hainan Province, the South China Sea. It was preserved in 95% ethanol and deposited in Hainan Academy of Ocean and Fisheries Sciences (Jin Gao, gaojin427@126.com) with voucher number 20200416YP08. Total genomic DNA was extracted from the muscle by using DNeasy Blood & Tissue (Qiagen, Valencia, CA). The extracted DNA was sequenced through high-throughput Illumina Novaseq platform (Total Genomics Solution Limited, SZHT). We performed *de novo* assembly by using the GetOrganelle v1.6.2e (Jin et al. 2020) to produce the complete mitogenome. All genes were identified using BLAST search in NCBI or tRNAscan-SE search server (Schattner et al. 2005) and annotated using the software of MITOS (Bernt et al. 2013).

The complete mitogenome of *A. trimaculatus* (GenBank accession number: MZ411565) is 16,548 bp in size, containing 13 protein-coding genes (PCGs), 22 tRNA genes, two rRNA genes (12S and 16S), and a non-coding control region of D-loop. Among these 37 genes, nine genes (*nad6*, *tRNA^Gln^*, *tRNA^Ala^*, *tRNA^Asn^*, *tRNA^Cys^*, *tRNA^Tyr^*, *tRNA^Ser^*, *tRNA^Glu^*, *tRNA^Pro^*) are encoded and located at the light (–) strand, while the remaining 28 genes are on the heavy (+) strand. Among the 13 PCGs, most of them use ATG as the initiation codon except *cox1* (with GTG). Besides, nine PCGs use TAA as the stop codon, but *nad2*, *cox3*, *nad3*, and *nad6* terminate with TAG codon. The TAA stop codon in total having five genes (*cox2*, *cox3*, *nd3*, *nd4*, and *cytb*) is completed by the addition of 3′ A residues to the mRNA. The overall nucleotides composition is 28.4%, 25.6%, 29.5%, and 16.5% for A, T, C, and G, respectively, with a slight high A + T content (54.0%).

In order to validate the phylogenetic position of *A. trimaculatus*, a maximum-likelihood phylogenetic tree was constructed using MEGA7.0 (Kumar et al. 2016) with 1000 bootstrap replicates (Figure 1) based on *A. trimaculatus* and other 12 species derived from different genus within Pomacanthidae. *Scomber australasicus* derived from Scombridae was selected as the outgroup for tree rooting. The result showed that Apolemichthys was divided into two clusters with a bootstrap probability of 100% (Figure 1). Furthermore, *A. trimaculatus* had a closest relationship with *Apolemichthys armitagei* in the same clade. In conclusion, the *A. trimaculatus* mitogenome provided useful genetic information and an important dataset for future taxonomic and phylogenetic analysis of marine angelfish.

**Figure 1.**
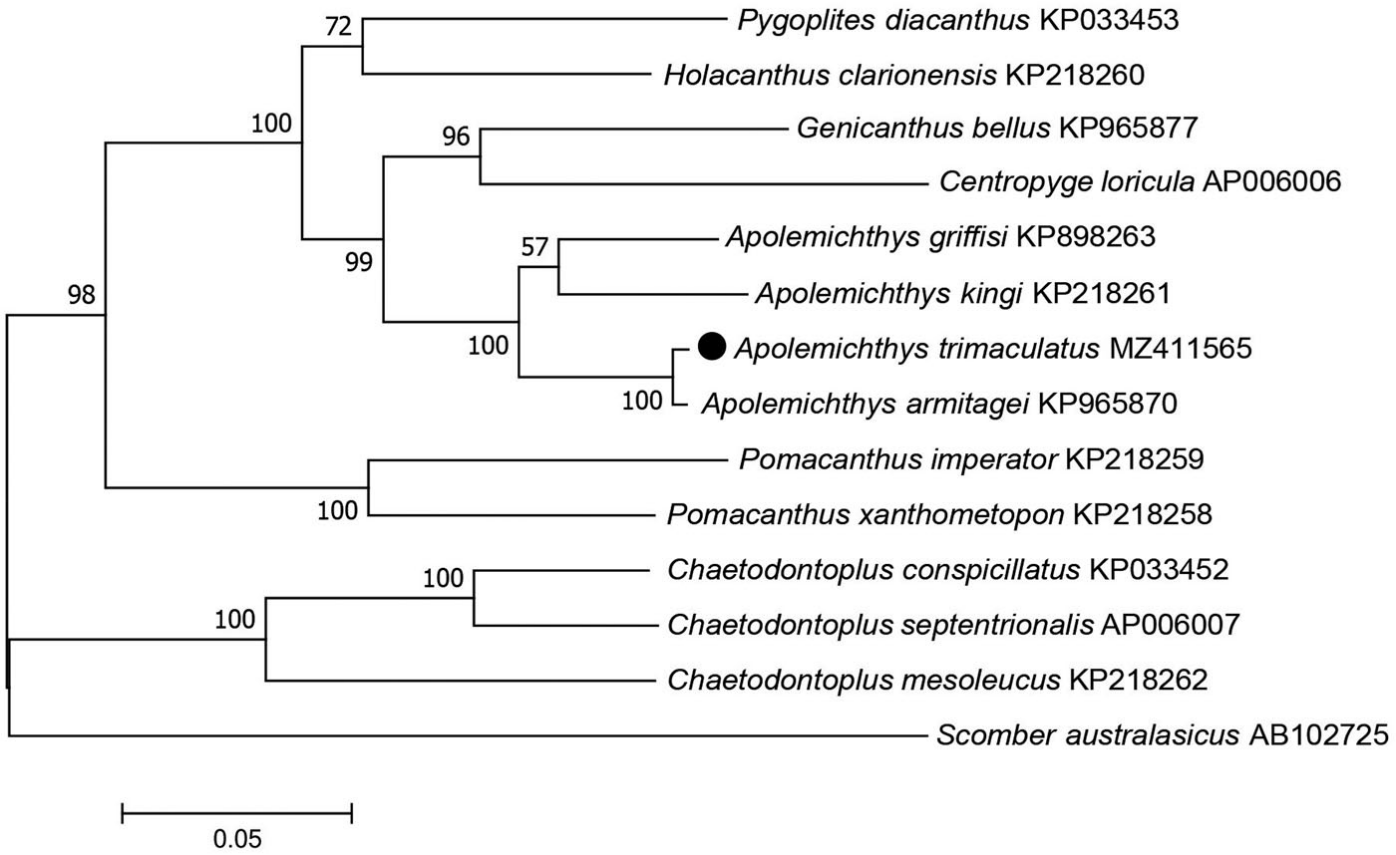
Phylogenetic tree of the complete mitogenome sequences of 13 fish species in Pomacanthidae. GenBank accession numbers of each sequence were listed in the tree with their corresponding species names. The mitogenome sequence in this study is labeled with a black dot.

## Data Availability

The genome sequence data that support the findings of this study are openly available in GenBank of NCBI at https://www.ncbi.nlm.nih.gov/ under the accession no. MZ411565. The associated BioProject, SRA, and Bio-Sample numbers are PRJNA748870, SRR15213040, and SAMN20346349, respectively.
